# Anti-inflammatory activity of standardized dichloromethane extract of *Salvia connivens* on macrophages stimulated by LPS

**DOI:** 10.1080/13880209.2017.1305423

**Published:** 2017-03-27

**Authors:** Marco Martín González-Chávez, Cinthia Saraí Ramos-Velázquez, Roberto Serrano-Vega, Cuauhtemoc Pérez-González, Ernesto Sánchez-Mendoza, Salud Pérez-Gutiérrez

**Affiliations:** a Facultad de Ciencias Químicas, Centro de Investigación y Estudios de Posgrado, Universidad Autónoma de San Luis Potosí, San Luis Potosí, SLP, México;; b Departamento de Sistemas Biológicos, Universidad Autónoma Metropolitana – Xochimilco, Ciudad de México, México

**Keywords:** Ursolic acid, HPLC, cellular viability, cytokine determination, eupatorin

## Abstract

**Context:** A previous study demonstrated that the chloroform extract of *Salvia connivens* Epling (Lamiaceae) has anti-inflammatory activity.

**Objective:** Identification of the active components in the dicholorometane extract (DESC), and, standardization of the extract based in ursolic acid.

**Material and methods:** DESC was prepared by percolation with dichlromethane and after washed with hot hexane, its composition was determined by CG-MS and NMR, and standardized by HPLC. The anti-inflammatory activity was tested on acute TPA-induced mouse ear oedema at doses of 2.0 mg/ear. The cell viability of macrophages was evaluated by MTT method, and pro- and anti-inflammatory interleukin levels were measured using an ELISA kit.

**Results:** Ursolic acid, oleanolic acid, dihydroursolic acid and eupatorin were identified in DESC, which was standardized based on the ursolic acid concentration (126 mg/g). The anti-inflammatory activities of DESC, the acid mixture, and eupatorin (2 mg/ear) were 60.55, 57.20 and 56.40% inhibition, respectively, on TPA-induced ear oedema. The IC_50_ of DESC on macrophages was 149.4 μg/mL. DESC (25 μg/mL) significantly reduced TNF-α (2.0-fold), IL-1β (2.2-fold) and IL-6 (2.0-fold) in macrophages stimulated with LPS and increased the production of IL-10 (1.9-fold).

**Discussion:** Inflammation is a basic response to injuries, and macrophages are involved in triggering inflammation. Macrophage cells exhibit a response to LPS, inducing inflammatory mediators, and DESC inhibits the biosynthesis of the pro-inflammatory and promote anti-inflammatory cytokines.

**Conclusion:** DESC has an anti-inflammatory effect; reduced the levels of IL-1β, Il-6 and TNF-α; and increases IL-10 in macrophages stimulated with LPS. Ursolic acid is a good phytochemical marker.

## Introduction

Inflammation is a response to an injury as part of the body’s immune response (Weiss 2008; Ashlet et al. 2012). Initially, this process is beneficial because it is a part of the healing process; however, inflammation can occasionally cause further inflammation. Non-steroidal anti-inflammatory drugs (NSAIDs) and corticosteroids are used for the treatment of inflammation, but these drugs have severe side effects (Piper et al. 1991; Meck et al. 2009). Therefore, the search for new compounds with anti-inflammatory activity is ongoing, and plants are an important source of anti-inflammatory compounds.

The genus *Salvia* (Lamiaceae) includes more than 900 species. Several plants from this genus have been used for their beneficial healing properties (Rivera et al. 1994). Some secondary metabolites, such as essential oils, terpenoids, and phenolic compounds, have been isolated from this genus (Rodríguez-Hahn et al. 1992). One species of this genus is *Salvia connivens* Epling, which is a small shrub with blue flowers used to treat sore throat, fevers, diarrhoea, inflamed eyes, malaria, and also acts as an antipyretic agent. Pérez et al. (2009) reported that the chloroform extract of *S. connivens* possesses anti-inflammatory properties; nevertheless, there are no published phytochemical studies.

In this study, ursolic acid, oleanolic acid, dihydroursolic acid and eupatorin were identified in the dichloromethane extract of the leaves of *Salvia connivens* (DESC). These compounds possess significant anti-inflammatory activity (Baricevic et al. 2001; Chi-Rei et al. 2011; Laavola et al. 2012). Consequently, the present study performs a phytochemical study of DESC, standardizes DESC based on the ursolic acid concentration using HPLC analytical technique, and determines the anti-inflammatory activity of this extract on ear edema in mice induced with TPA and its effect on murine macrophages stimulated with lipopolysaccharide (LPS).

## Materials and methods

## Plant material

Fresh aerial parts of *S. connivens* were collected from the Las Comadres municipality of Guadalcazar, San Luis Potosi State, Mexico, in July 2012, and the plant was identified by the taxonomist José Pérez. A voucher specimen (SLPM43013) was deposited in the Herbarium Isidro Palacios of the Universidad Autónoma de San Luis Potosí.

## DESC preparation

Dried, ground aerial parts (100 g) were extracted by percolation with dichloromethane and washed with hot hexane. The solvent was then evaporated to dryness in a rotatory evaporator under reduced pressure; the yield was 5.1%.

## DESC fractionalization

The extract (5 g) was separated by column chromatography. The column was packed with silica gel (Merck 60, 70-230 mesh ASTM), which was prepared using hexane as the mobile phase. Then, the polarity was increased with ethyl acetate. Fractions of 100 mL were collected and compared by thin-layer chromatography; fractions with the same chromatographic pattern were pooled. The resulting fractions were tested on ear oedema in mice induced by 12-*O*-tetradecanoylphorbol-13-acetate (TPA), and the composition of the fractions with anti-inflammatory activity was determined.

## Chemical composition of the active fractions

Fraction 7, which was obtained with hexane/ethyl acetate (FHA) (90:10 v/v), produced a white solid (465 mg). Prior to the GC-MS analysis, the mixture was derivatized into trimethylsililethers as follows: 7 mg of the solid or 2 mg of the standard samples were dissolved in 2 mL isooctane. For trimethylsilylation, 100 μL of N,*O*-bis(trimethylsilyl)trifluoroacetamide with 10% of trimethylchlorosilane was added to the samples and heated to 100 °C for 10 min in a CEM Discover microwave at 150 W and 290 psi (Matthews, NC).

GC-MS analysis was performed on a gas chromatograph coupled to a mass spectrometer (Agilent Technology, model 6890 N, Santa Clara, CA), which was coupled to a mass selective detector (model 5973) with an HP-5MS capillary column (30 m in length, 0.25 mm internal diameter and 0.25 μm film thickness). The injector temperature was 250 °C. This temperature program started at 150 °C for 2 min with a heating rate of 15 °C/min up to 250 °C; this temperature was maintained for 1 min. A splitless injection was performed at a ratio of 1:100, and the injector temperature was 320 °C. The spectra were measured at 70 eV. The compounds were identified based on their mass spectra by comparison to the spectra of the standard samples.

**Figure 1.**
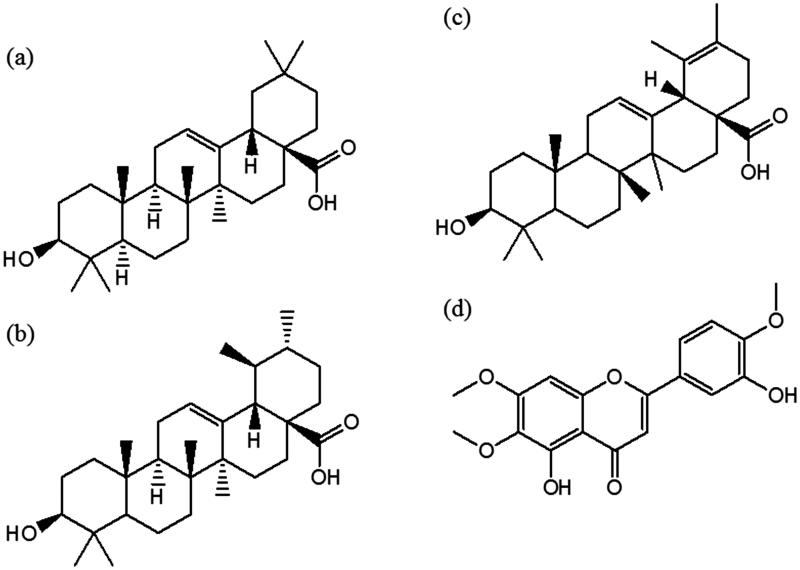
Structures of (a) ursolic acid, (b) oleanolic acid, (c) dihydroursolic acid, and (d) eupatorin.

After derivatization, the white solid was identified as a mixture of three compounds, which were the following trimethylsililethers:

41.8% oleanolic acid (Figure 1(a)). MS (EI): *m/z* (%): 600 (M^+^, 2.8), 585 (6.6), 482 (21.7); 320 (31.1), 279 (4.7), 203 (100.0), (35.8), 133 (54.9), 73 (80.4);
47.69% ursolic acid (Figure 1(b)). MS (EI): *m/z* (%): 600 (M^+^, 2.0), 585 (8.8), 482 (15.7), 320 (93.1), 279 (11.8), 203 (100.0), (40.2), 133 (54.9), 73 (80.4); and
11.13% dihydroursolic acid (Figure 1(c)). MS (EI): *m/z* (%): 598 (M^+^, 11.6), 583 (3.9), 481 (12.6), 305 (14.6), 201 (41.7), (36.9), 73 (100.0).

A yellow solid was obtained from fraction 13 with hexane/ethyl acetate (90:10 v/v; Perkin Elmer Paragon 1600, Billerica, MA) and identified as eupatorin (Figure 1(d)): 76.5 g; m.p. 194 °C; IR (KBr) ν_max_ = 3433, 3070, 2945, 1652, 1603, 1271, 841 cm^−1^; ^1^H-NMR; Agillent NMR Spectrometer DD2, Palo Alto, CA (600 MHz, CDCl_3_): δ 3.73 (s, 3 H), 3.86 (s, 3 H), 3.91 (s, 3 H), 6.76 (s, 1 H), 6.86 (s, 1 H), 7.07 (d, 1 H, *J*o = 9 Hz), 7.44 (d, 1 H, *J*m = 2.4 Hz), 7.54 (dd, 1 H, *J*o = 8.4 y *J*m = 2.4 Hz), 12.87 (s, 2 H). ^13^C-NMR (150 MHz), δ: 56.12, 56.66, 60.28, 91.99, 103.75, 105.59, 112.65, 113.72, 119.49, 123.70, 132.61, 147.71, 152.26, 152.99, 153.62, 159.57, 164.79, 183.15. MS (EI): *m/z* (%): 344 (M^+^, 100), 329 (88.9), 315 (24.7), 298 (24.7), 153 (24.7).

## Standardized extract

The DESC was analyzed by HPLC (Varian Model Pro Star 310, Palo Alto, CA) with an Evaporative Light Scattering Detector (ELSD) (Alltech model ELSD 2000; Bath, UK) using N_2_ with a flow of 1.7 L/min at 67 °C evaporation temperature. The chromatographic conditions were evaluated and optimized in a Grace Smart RP C18 column (5 μm, 4.6 × 250 mm). The column temperature was 40 °C. The flow rate was set to 0.7 mL/min. The linear gradient elution of the mobile phase was initiated with 0.5% (v/v) acetic acid in a water-methanol-acetonitrile solution with a ratio of 5:30:65 (v/v) and completed after 20 min with a composition of 0.5% acetic acid in a water-methanol-acetonitrile solution with a ratio of 5:10:85 (v/v). The curve calibration was realized with six points (concentrations of 0.06, 0.12, 0.18, 0.24, 0.30 and 0.60 mg/mL) of ursolic acid standard (Sigma-Aldrich, México, Toluca, Edo Mex). Each point was obtained from the average of three injections; the volume was adjusted to 20 μL for all samples. The integral chromatographic peak, which was obtained at 6.21 min of retention time, was used to generate the standard calibration curve (*R* ^2^ = 0.99637). DESC (6 mg) was dissolved in 1 mL of methanol (0.6 mg/mL), and 20 μL of the sample were injected by triplicate.

## Animals

Male CD-1 mice (20–25 g) from the Universidad Autónoma Metropolitana-Xochimilco Animal Facility were housed in isolated cages at 24 °C under 12 h light/dark cycle. The animals were supplied with Lab Diet 5001 food and water *ad libitum*. The experiments were conducted according to the Official Mexican Norm NOM 062-ZOO-1999 (technical specifications for the production, care and use of laboratory animals). All animal procedures were approved by the Research Bioethics Committee of Universidad Autónoma Metropolitana-Xochimilco.

## TPA-induced mouse ear oedema

The method for TPA-induced mouse ear oedema has been described previously (Young de et al. 1989). A solution of TPA (2.5 μg) in acetone (25 μL) was topically applied to the inner and outer surfaces of the right ears of mice, and acetone alone was applied to both surfaces of the left ear. Thirty minutes later, 2.0 mg/ear of DESC, fraction 7, fraction 13 or IND dissolved in acetone was topically applied to the right ear, and acetone was applied to the left ear. After 6 h, the animals were sacrificed, and 6 mm plugs of the central portion of both ears were weighed. The percent inhibition of oedema was determined.

## Cell viability assay

J774A.1 macrophages were washed with a PBS buffer. The solution was decanted, and the cells were seeded in a Dulbecco’s Modified Eagle’s Medium (DMEM) in 96-well microplates at a density of 8 × 10^4^ cells per well and incubated for 24 h. Next, the extract was dissolved in DMSO at a concentration of 1 to 200 μg. After 48 h of treatment, 10 μL of 3-(4,5-dimethylthiazol-2-yl)-2,5-diphenyl tetrazolium bromide (MTT) at 5 mg/mL in a PBS buffer were added, and the plates were incubated for 4 h at 37 °C. Then, the medium was removed, and the formazan crystals were dissolved in 100 μL of DMSO. The optical density (OD) was determined at 540 nm in an ELISA plate reader from BioRad. Six replicate wells were used to determine the viability using the following equation.

$$\%\;Viability=\frac{\;({OD}_{treated\;cells})}{{OD}_{control\;cells}}\times 100$$

## Determination of pro- and anti-inflammatory interleukin levels

TNF-α, IL-6, IL-10, and IL-1β serum levels were measured using a commercially available ELISA kit according to the manufacturer’s instructions. The OD was measured in a microplate reader at 405 nm with a wavelength correction set at 650 nm.

J774A.1 macrophages were grown at a density of 1 × 10^6^. Then, the cells were treated separately with the DESC at a concentration of 25 μg/mL, whereas another group was treated with IND at a concentration of 50 μM. The cells were incubated for 2 h. After treatment with LPS (1 μg/mL) and incubated for 24 h, the cell-free supernatants were collected and stored at −20 °C until they were analyzed by immunoassay for the quantification of cytokines. Specifically, the concentrations of IL-6, IL-1β, IL-10 and TNF-α in the supernatants of the J774A.1 cell culture were determined using a commercial immunoenzymatic kit PEPROTECH (Rocky Hill, NJ). 

## Statistical analyses

The results are expressed as the means ± SEM. Statistical analyses were performed using Student’s *t*-test and ANOVA, which was followed by Tukey’s test. Statistical significance was set at *p* < 0.05.

## Results

The white solid obtained in fraction 7 was identified by GC-MS analysis as a mixture of the following three compounds: oleanolic acid (41.18%), ursolic acid (47.69%) and hydroursolic acid (11.13%) (Figure 1, structures a, b, and c, respectively).

Fraction 13 contained a yellow solid that was identified by ^1 ^H-and ^13 ^C NMR, MS and IR spectroscopy analysis (summarized in the ‘Materials and methods’ section) as the flavonoid eupatorin (Figure 1(d)). The chemical shifts were similar to the signals of the same flavonoid previously isolated from *Lantana montevidensis* (Spreng) Briq. (Nagao et al. 2002) and *Sideritis bolleana* Bornm. (González et al. 1978).

**Figure 2.**
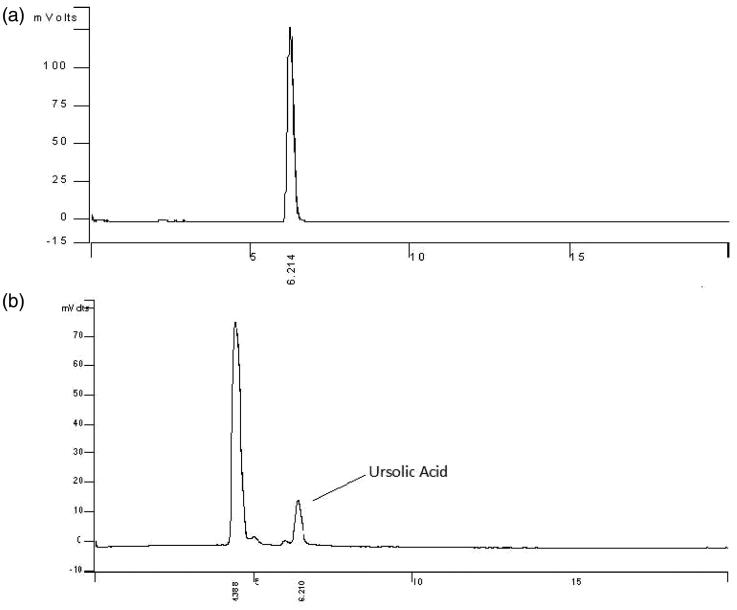
Chromatograms of the ursolic acid reference (a) and DESC solution (b).

The DESC was standardized using ursolic acid as phytochemical marker. This compound displayed a retention time of 6.1 min; a representative chromatogram of ursolic acid and DESC is presented in Figure 2. The calibration curve was linear in the 0.06 to 0.6 mg/mL range (*R* ^2^ = 0.99637). The results showed that the DESC contains 126 mg/g (12.6%) of ursolic acid.

DESC, the acids mixture and eupatorin at doses of 2 mg/ear reduced TPA-induced ear oedema by 60.55 ± 1.8%, 57.2 ± 1.1% and 56.4 ± 1.4%, respectively. The inhibitory activities of the three of them were similar to that observed when IND at a dose of 2 mg/ear IND (64.5 ± 1.4%).

**Figure 3.**
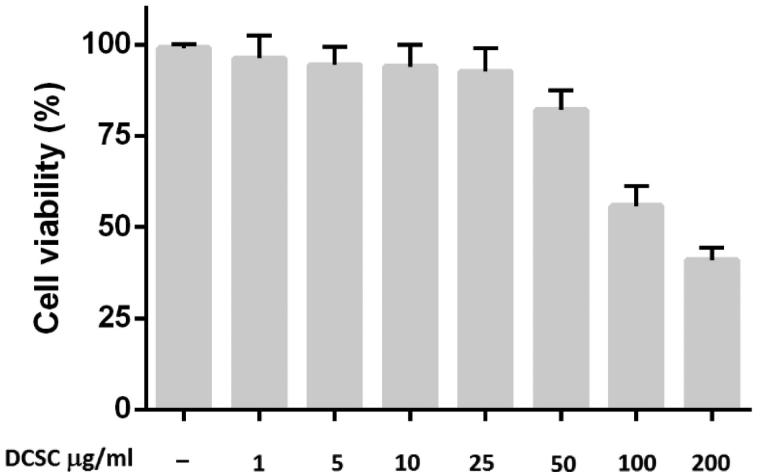
Cell viability in macrophages treated with DESC at 1, 5, 10, 25, 50, 100 and 200 μg/ml, as determined by the MTT assay. Results are expressed as the percentage of surviving cells relative to control cells. The results are the mean of three determinations ± SEM.

**Figure 4.**
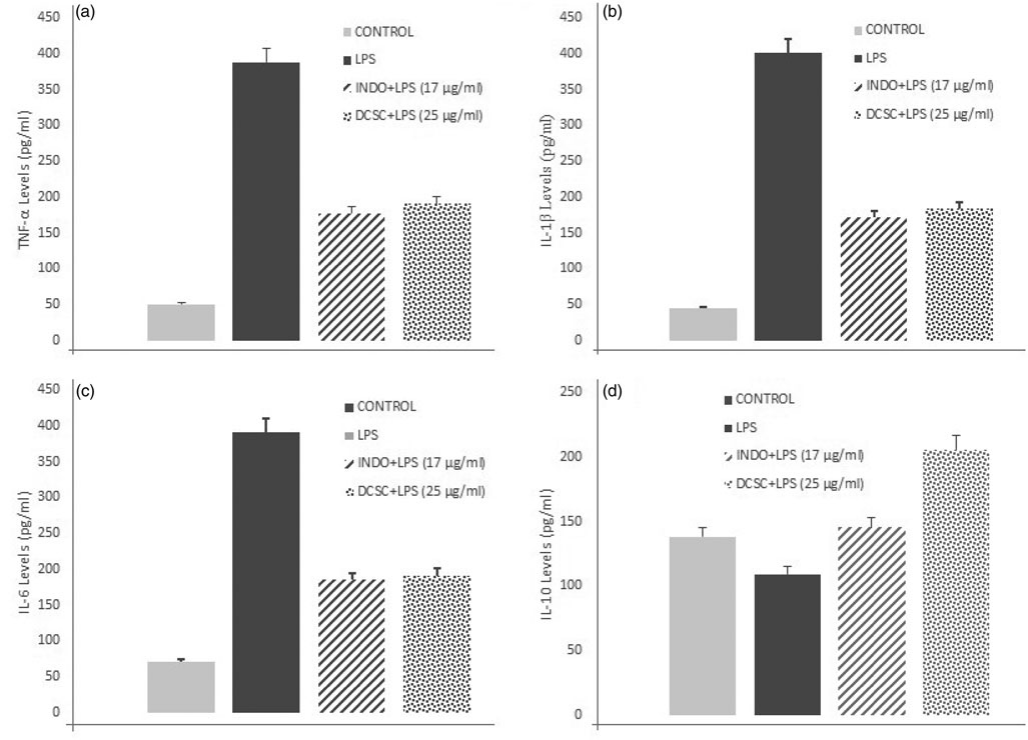
Effects of the DESC extract on the LPS-stimulated levels of (a) TNF-α, (b) IL-1β, (c) IL-6 and (d) IL-10 levels in macrophages. The concentrations were determined by ELISA. The results are the mean of three determinations ± SEM. * *p* < 0.05 vs. LPS group.

The cell viability of J774A.1 macrophages was evaluated. Figure 3 shows that DESC at concentrations of 1, 5, 10, 25 and 50 μg/mL, did not affect cell viability, whereas 100 and 200 μg/mL DESC did affect cell viability. Therefore, we used 25 μg/mL of DESC in subsequent experiments. The IC_50_ was found as 149.4 μg/mL. The effect of DESC and IND on the production of the pro-inflammatory cytokines TNF-α, IL-1β and IL-6 and the anti-inflammatory cytokine IL-10 was measured in the culture medium of macrophages stimulated with 1 μg/mL of LPS alone or in combination with 25 μg/mL of DESC (Figure 4). DESC significantly reduced TNF-α (2.0-fold), IL-1β (2.2-fold) and IL-6 (2.0-fold), and the effects were similar to those obtained with IND. Moreover, DESC increased IL-10 production (1.9-fold) at the same concentration compared with the control group, and the increase in IL-10 production was higher than that observed in the group treated with IND.

## Discussion

The EI-MS fragmentation patterns of the compounds of the white solid obtained from fraction 7 were similar to those obtained with the reference samples of oleanolic acid, ursolic acid and hydroursolic acid. The anti-inflammatory properties of oleanolic and ursolic acids have been previously reported (Huang et al. 1994; Liu 1995; Vasconcelos et al. 2006).

The inhibitory activities of DESC, acids mixture and eupatorin were similar to that observed when IND at a dose of 2 mg/ear was tested (64.5 ± 1.4%). Thus, we suggest that the anti-inflammatory effect of DESC is due to ursolic acid (Baricevic et al. 2001), oleanolic acid (Chi-Rei et al. 2011) and eupatorin (Laavola et al. 2012), which has been previously reported.

Ursolic acid suppresses the expression of COX-2 and iNOS (Suh et al. 1998), inhibits the production of NO (Ryu et al. 2000), and activates NF-кB production (Shishodia et al. 2003). For these reasons, we considered ursolic acid an appropriate phytochemical marker for use in developing a method for standardizing DESC.

The effect of DESC and IND on the production of the pro-inflammatory cytokines TNF-α, IL-1β and IL-6 and the anti-inflammatory cytokine IL-10 was measured in the culture medium of macrophages stimulated with 1 μg/mL of LPS alone or in combination with 25 μg/mL of DESC (Figure 4). DESC significantly reduced TNF-α (2.0-fold), IL-1β (2.2-fold) and IL-6 (2.0-fold), and the effects were similar to those obtained with IND. Moreover, DESC increased IL-10 production (1.9-fold) at the same concentration compared with the control group, and the increase in IL-10 production was higher than that observed in the group treated with IND.

Inflammation is a basic response to injuries and protects the body from inflammatory stimuli, and macrophages are involved in triggering inflammation in pathological conditions. Macrophage cells exhibit a response to LPS, which is an endotoxin that induces a variety of inflammatory mediators, such as TNF-α, IL-1β and IL-6 (Rankin 2004). Thus, therapeutic agents that inhibit the biosynthesis of the pro-inflammatory mediators and promote anti-inflammatory cytokines may be useful in treating inflammatory conditions.

## Conclusions

DESC has an anti-inflammatory effect; reduced the levels of IL-1β, Il-6 and TNF-α; and increases the production of IL-10 in macrophages stimulated with LPS. One of the compounds responsible for this activity is ursolic acid, which was used for DESC standardization by HPLC. This method was adequate.
